# Bridging the genomic data gap in Africa: implications for global disease burdens

**DOI:** 10.1186/s12992-022-00898-2

**Published:** 2022-12-09

**Authors:** Olabode Ebenezer Omotoso, John Oluwafemi Teibo, Festus Adebayo Atiba, Tolulope Oladimeji, Ayomide Oluwadarasimi Adebesin, Ahmad O. Babalghith

**Affiliations:** 1 Department of Biochemistry, College of Medicine, University of Ibadan, Ibadan, Nigeria; 2 Department of Biochemistry and Immunology, Ribeirão Preto Medical School, University of São Paulo, Ribeirão Preto, São Paulo, Brazil; 3 Department of Zoology, University of Ibadan, Ibadan, Nigeria; 4 Department of Biochemistry, Cancer Genomics Lab, Covenant University, Ota, Nigeria; 5 Medical Genetics Department, College of Medicine, Umm al-qura University, Makkah, Saudi Arabia

**Keywords:** Genomics, Data gap, Genetic repositories, Global disease burden, Africa

## Abstract

This paper highlights the gap in the use of genomic data of Africans for global research efforts for disease cures. Genomic data represents an important tool used in disease research for understanding how diseases affect several populations and how these differences can be harnessed for the development of effective cures especially vaccines that have an impact at the genetic level e.g., RNA vaccines.

This paper then provides a review of global genomic data status where three continents are reported to be the major contributor of genomic data to repositories used for disease research and the development of vaccines and medicines around the world.

We reviewed the most recently published information about genetic data inclusiveness of populations, explaining how genomic data of Africans is lacking in global research efforts that cater towards the eradication of pandemics via the development of vaccines and other cures. We also discuss the implication of this non-inclusiveness for global disease burdens and indicate where changes need to be made in the last part of the paper.

Lastly, the entire centers on some general policy recommendations to fully include African genomic data in such global genetic repositories. These recommendations can be implemented in African countries to improve genetic data collection, storage, and usage policies.

## Introduction

Diseases continue to impact human lives today in many ways. Beyond public health burdens, they present nations, communities, families, and individuals with excruciating social, economic, and financial burdens. However, the ripple effect of these burdens has galvanized global efforts to tackle the diseases, albeit lopsidedly. As of June 2022, the global population is estimated to be around 7.96 billion. About 17% (1.34 billion) of the global population live in Africa, second only to Asia’s 61 % (or 4.7 billion people) [1]. Africa is also projected to have the highest population growth globally, with the population doubling between now and 2050 [2].

Aside from Africa’s high population trends, reports [3–5] indicate that Africans have the most diverse population genetics as it is regarded as the epicenter of modern human origin [6]. Despite this, only a small percentage of African genomic data is available to contribute to the ongoing efforts toward global disease prevention and management, thus creating a genomic data gap [7]. A genomic data gap is defined as “the (intentional or unintentional) omission of genomic data of a group or subset of a population by researchers in a scientific effort or research, which requires inclusive genomic data. e.g., population studies that involves people of all major ethnic divisions or countries.”

In most recent advances or decisions in science, especially precision medicine and vaccine development, Africa has been left out, with genetic information available from American, European, and Asian populations [8]. But why does this matter, if current remedies for diseases (e.g., COVID-19) are effective for almost every population around the world, even though genetic information is gotten from a few subsets of the global human population? One interesting implication is efficacy - cures that are effective in a particular population and less effective in others [6]. Hence, unmasking Africa’s rich genomic spectra would improve our understanding of the genetic basis of single- and multi-gene disease burdens, which would go a long way in improving global disease research efforts.

**Fig. 1:**
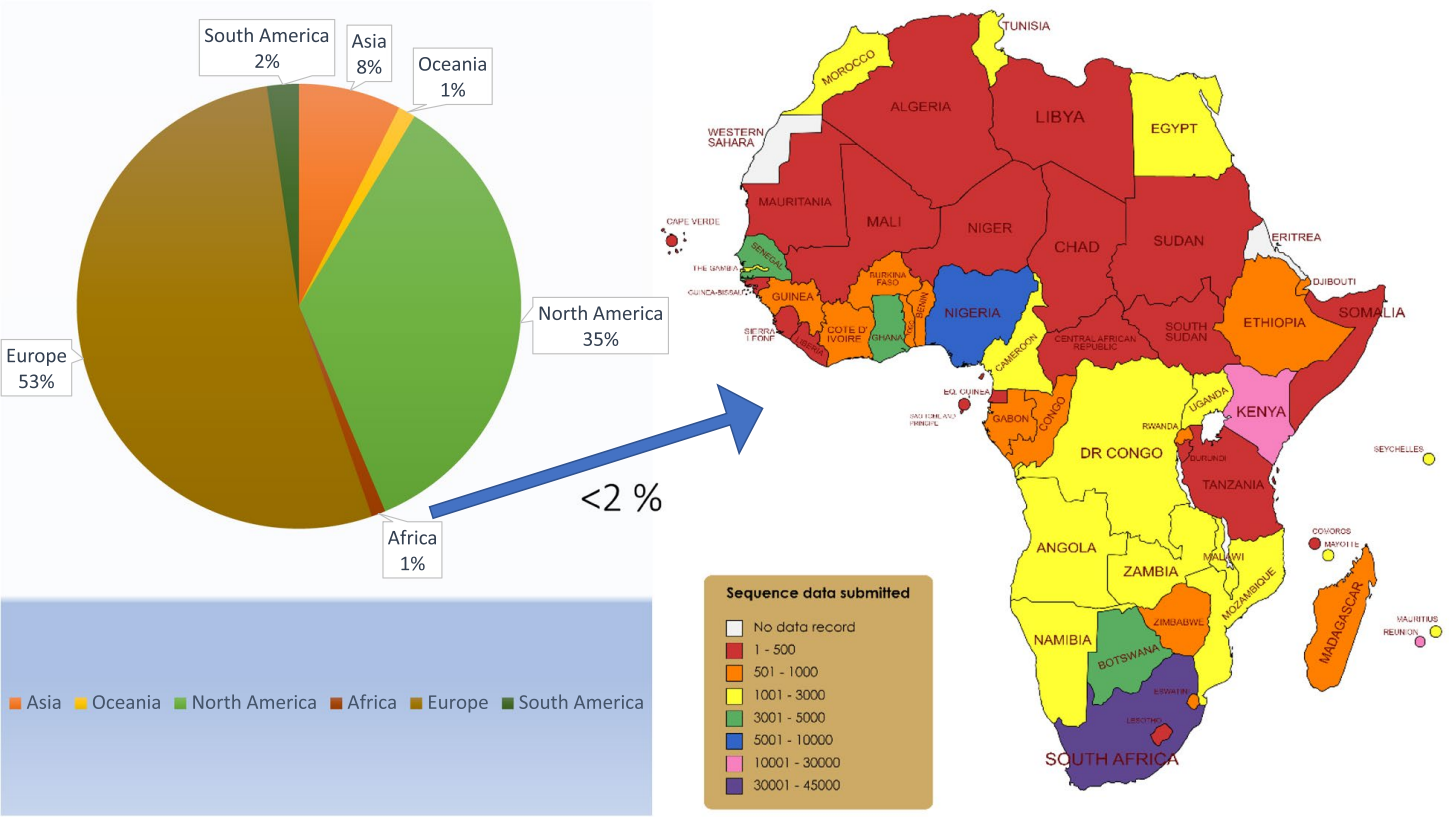
Comparative chart (inter- and intra-continental) of the number of SARS-CoV-2 viral sequences deposited in the GISAID. Africa has contributed the lowest number of sequence data generated compared to other continents. Most countries generated few sequences (< 500), while South Africa deposited close to 40% of all data generated in Africa

The completion of the Human Genome Project (HGP) between 1990 and 2003 [9] elicited a new frontier in scientific approach to tackling diseases of public health concern. Discoveries from the HGP also contributed to the recent advances in our understanding of human evolution and disease transmission, public health genomics, forensics, anthropology, vaccine and drug development, targeted therapy, rare disease research, precision, and personalized medicine [3]. Because current efforts in medicine today are leaning towards precision/personalized medicine, genomic data has become more important for most disease prevention strategies. Therefore, it is expedient that genetic diversity is prioritized for equity of medical research in the global efforts to provide cures that cater to everyone and reduce disease burdens worldwide. Without this genomic data inclusiveness, there are grave implications for global health. For instance, variants from uncharacterized genetic mutations could hamper disease eradication. About 3.4 million distinct undocumented gene variants were reported in an analysis of about 400 human genomes from 13 African countries [10]. And as of January 2019, Africans only represent 3% of genome data used for genome-wide association studies (GWAS), but this figure has drastically reduced to 1.1% in 2021 owing to several factors such as lack of infrastructure and enabling environment for genomic studies, scarce or no funding and politics. Similarly, people of African descent only represent 1.6% of the genotype data of 487,000 individuals in the U.K. Biobank resource [11]. This gap in genomic sequencing capability also became glaring during the COVID-19 pandemic, as Africans only contributed barely 2% of the total sequence data generated [12]. This is shown in Fig. 1 from data deposited in the GISAID [13]. Interestingly, about half (51%) of these data were generated from just three countries (South Africa, Kenya, and Nigeria) [14].

Other contributory factors to a lack of appropriate representation in genetic diversity in human genetic studies and disease research include low or no demand for research output, high cost and logistics challenges in sourcing research items, exploitation and misuse of African data, reliance on genomic data from Africans in diaspora [15].

Despite these shortcomings, some new initiatives have made giant strides in the growth of genomics in Africa. Among others, these initiatives include 54Gene [16], The African Centre of Excellence for Genomics of Infectious Diseases (ACEGID) [17], Inqaba Biotec (Africa’s Genomics Company) [18], and The Human Heredity and Health in Africa (H3Africa) Consortium [19] - a genomics research foundation in Africa with funding from the United States National Institutes of Health, the U.K.’s Wellcome Trust and the African Society of Human Genetics (AfSHG). H3Africa employs African investigators to determine genomic and environmental determinants of common diseases [20]. The 54Gene pioneered the Non-Communicable Diseases Genetic Heritage Study (NCD-GHS) consortium, based in Nigeria, which aims to assess the burden and etiological characteristics of non-communicable diseases in 100,000 Nigerians (spanning about 300 different ethnicities) to produce a comprehensive catalogue of human genetic variation among Nigerians [11]. Inqaba Biotec, with subsidiaries across sub-Saharan Africa, offers genomics products and services for researchers in Africa. Recently, the H3Africa consortium, the Collaborative African Genomics Network (CAfGEN), was established to address pediatric HIV and HIV-TB disease progression via genomic data gathering and analysis [21]. All of these are commendable developments, but a lot still needs to be done to bridge this gap.

Since it has been established that the genomic data encoded in Africa (ns) is waiting to be mined, we must take swift action. Bridging the genomic data gap in Africa would entail a multidisciplinary approach to improve research capacity through strategic funding and collaborations with existing and emerging genomic leaders as well as stakeholders in the field of genomics. A template for such action has been recently reported [11]. In the text above, we described the current standpoint, genomic data gap and other challenges impeding advances in genomics research in Africa. We then highlight the implications of a lack of genetic data inclusion for global disease burdens as well as provide recommendations on effective ways to bridge the identified gaps.

## Implications of African genomic data gap on global disease burden

The completion of the Human Genome Project in 2003 served as the frontier for the advancement of genomics and the progression of genetic research for disease studies [22]. Genomics has become increasingly important in clinical and public health research. For instance, genomics tests are now widely and frequently used in medical practice including non-invasive prenatal screening, analysis of genome tumors, and understanding the genomic background of different diseases. Other instances of importance include environmental genetics and how it contributes to disease emergence, its intervention, and research for cure [23]. Some of the followings are implications for global health due to the lack of inclusiveness in the currently available cures and research efforts:

### Unreported disease variants and drug resistance

The world’s population current figures include Asia – 59.5%, Africa – 17.2%, Europe – 9.6%, South America (Latin America and the Caribbean) – 8.4%, North America – 4.8%, and Oceania – 0.5% [1]. Despite this, therapies and pharmaceuticals developed to tackle infectious and non-communicable diseases are predominantly of European origin, clinically tried on a less diverse (genetic) population but developed for global use. A case that typically depicts this pitfall is the 2016 scenario when approximately 13.5% of the population in Botswana was reported to have two copies of the gene variant, which was responsible for the slow metabolism of Efavirenz – a widely accepted therapy used in the region for HIV treatment [24]. Similarly, 14–34% of African descent were reported [25] to harbor an exclusive genetic variant in the gene encoding a liver metabolizing enzyme (CYP2D6), which reduces the efficacy of the breast cancer chemotherapeutic agent, Tamoxifen. If genomic studies in Africa - the most genetically diverse population of humans - are not prioritized, global health equity would be hampered.

Over the years, high-depth whole genome sequence data has revealed more than 3 million novel and previously unreported variants from only about 400 Africans [26]. However, despite the growth of data in genomic repositories [27], several of these unreported variants from the Africans are not included. This lack of inclusion has been implicated in several environmental adaptation, ineffective cures, and disease susceptibility. For instance, the underrepresentation of African diversity in preclinical drug development due to the genomic data gap was also implicated [28] as a driving force for a high prevalence of severe adverse drug reactions (ADRs) to Cytochrome P450-Mediated Drug Metabolism amongst Africans. Another study carried out on type 2 diabetes in population around sub-Saharan Africa discovered a new type of gene family - RanBP2-type ZnFs [29]. If this trend continues unaddressed, it may decrease adherence to treatment regimen, and promote vaccine evasion and drug resistance. This underrepresented population can serve as human reservoir of some lethal diseases, which are likely to spread through migration and globalization. Hence, equitable representation of all human populations in genomic studies and disease research would give more insight into disease prevention and curative strategies, benefiting the world.

### Quality of research and efficacy of cures

Africa is vastly underrepresented in genetic research. Only a small proportion of African genomic data is readily available to contribute to disease prevention, detection, and treatment strategies [30]. For instance, as of January 2019, just 3% of the genomic data used in genome-wide association studies (GWAS) are from Africans [15], which reduced drastically to 1.1% in 2021 [31]. Majority of genomic datasets used come from Europe, Australia, and North America despite the enormous population of Africa, its evolutionary history, and genetic diversity. The absence of genomic data in Africa thus restricts global healthcare and research efforts.

As earlier mentioned, African genetic diversity can give insight to unravel novel disease susceptibility, increase the chances of correct diagnosis and improve the chances of clinical trial success. It has been proven that the analysis of large-scale genomic data is a crucial component of precision medicine and has significant potential to inform clinical care. Fatumo et al. [31], demonstrated the potential of African genome as an excellent resource for genomic research and precision medicine by collecting and analyzing genome-wide data from 14,126 individuals from Uganda, South Africa, Kenya, and Nigeria. They found 10 new genetic variants associated with several traits or diseases, with 9 of them peculiar to the African population. These adverse impacts highlighted further reiterate why investments toward developing genomics research capacity in Africa is crucial. Teibo et al. [32], also reiterated the importance of African genomics in a recently published paper in Nature Africa. Globalization is increasing, therefore continental boundaries can no longer contain the extent of disease spread. The COVID-19 pandemic exemplifies this, proving how diseases can spread rapidly from a corner of the world and significantly influence human history globally – how we socialize, interact, travel, transact and even live. Other possible implications include concerns about increased chances of re-infection, human reservoirs of some lethal diseases and drawback on the recent advances in precision or personalized medicine.

## Policy recommendations for bridging the genomic data gap

Governments by far have the highest level of influence on society especially including decisions that could affect the socioeconomic and health outcomes of a population. Investments towards anything that could improve these outcomes are therefore important for any country. Since research is an important aspect of health, investing in genomics research in Africa can prove vital for the improvement of health globally. What this implies for global health inclusion of African genome data would improve precision medicine through solutions like vaccines and drug development, reduce the chances of disease re-infection, drug resistance, and even future pandemics. We suggest the following policy recommendations to bridge the genomic data gap in Africa.

### Building a representative genomic repository for Africans

Concerns about intercontinental genomics data inequalities had risen as far back as 2009 when it was reported [33] that 96% of participants in genome-wide association studies (GWAS) were of European descent. Africa has been established to be a genetically diverse continent representing 54 countries with over 3000 ethnic groups. However, much of its genomic data is attributed to Africans living in the diaspora, which is not an adequate representation of the diverse genome that span the continent [5]. Some of these diasporans are not fully African as they have deep interracial relations with people from other continents (e.g., children from interracial marriages). Some are often affected by the environment in which they live too. Hence, the possible influence of inter-racial relations and gene-environment interaction must be considered if we would get the best of African genomic data.

Now, organizations like the H3Africa, ACEGID, 54Gene, Institute of Human Virology, African Collaborative Center for Microbiome and Genomics Research, African Research Group for Oncology, and Center for Genomic and Precision Medicine, amongst others, have risen to fill this gap. Today, Nigeria, with one-fourth of the total African population is taking the lead in the generation of genomic data for the continent, as demonstrated by Fatumo et al.*,* 2022 [11]. However, African data in GWAS still accounts for < 1% [34]. To tackle this gross underrepresentation, local researchers, i.e., those domiciled within the continent, must be empowered with the funds and tools to contribute their quota to building a larger genomic data repository that can cater to the larger population without having to collect data abroad.

### Developing research capacity across countries on the continent

A key consideration in addressing this status quo is developing research capacities through improved research facilities and technical expertise in Africa. African researchers do not lack the knowledge or skill for this genomic revolution, rather, they lack the key infrastructure, strategic partnership, favourable policy, and sufficient funding, as earlier mentioned. Hundreds of African researchers have acquired knowledge and expertise in well-equipped laboratories and institutes overseas through funded graduate studies, postdoctoral fellowships, grants, and collaborations. The challenge is coming back home to find that there is little or no opportunities for (world-class) research and genomic infrastructure for them to contribute their expertise.

However, we can take a cue from what already exists and the difference that such facilities are contributing. The H3Africa project, in its 12 years of commencement, has spearheaded many breakthrough research findings in Africa, most of which cumulates to form a huge chunk of the < 1% African data in global GWAS. This was achieved through a $176 million grant provided by the U.S. National Institute of Health (NIH) and Wellcome Trust [19]. H3Africa has established its presence in 30 African countries and facilitated the following research [35]:

- the AWI-Gen research that revealed that hypertension is highly prevalent in Eastern and Southern Africa;
- the Genomic Characterization and Surveillance of Microbial Threats in West Africa, which led to the development of SHERLOCK- a low-cost genomics test to rapidly identify three types of fever - which was deployed during the Lassa fever outbreak;
- Immunoglobulin gene diversity in an African population and impact on antibody function in HIV infection, clinical and genetics studies of hereditary neurological disorders in Mali and many others.

The NIH has proved very instrumental in addressing the genomic data gap in Africa. Recently, it granted $46 million for Alzheimer’s Disease Genetic Study, which will involve the genetic study of 5000 Africans and 4000 African Americans, among others [36].

In Africa, the financial allocation to support education and scientific research is insignificant compared to the volume of work that needs to be done. The reason for this is not far-fetched: the burden of other competing needs in the region, inarticulate communication of research findings to a non-science audience to foster the understanding of the importance of science, and significantly, corruption and misappropriation of scarce resources. This has led to brain drain of the continent. The International Development Research Centre in 2011 [37] identified that there are more African scientists and engineers in the United States than in the entire continent; most of whom have migrated for career prospects, better working conditions, and academia. But some strides have been made on the continent too. In 2018, South Africa recorded the highest scientific expenditure - 0.83% of its GDP [38]. Because of this seemingly extraordinary spending compared to other African countries, the country was ranked the 53rd most innovative country in the world by the Global Innovative Index [39]. This reveals that there are willing and capable hands on the continent and, if given the needed support in research capacity and technology development, they can contribute significantly to global health equity.

For robust results, setting strong local research ethics, laws and agendas that are of priority to the continent should be the foundation and should serve as templates for building local research capacity. The approach of scientists from High-Income Countries (HIC) coming to conduct “extractive or helicopter” research in Africa has not proven sustainable due to conflict of interest, ethical issues, claims on patent and data ownership, and dissemination of findings [40]. Hence, building local research capacity in- and about Africa’s health priorities is important for acquiring genomic data on the continent. However, local researchers should consider forming a partnership with HIC collaborators who understand the context and needs of the African region, teach agenda-setting skills, and assist in agenda development. African governments should also implement local specialized funding bodies seeking to finance research of African origin and relevance. Currently, the most frequent sponsors of research in Africa include the National Research Foundation and Medical Research Council in South Africa, the U.K. Department for International Development, The Bill & Melinda Gates Foundation, the German research-funding organization DFG, the WHO, the World Bank, USAID, NZAID, AUSAID, NIH, and Wellcome Trust [41, 42]. While these contributions have significantly advanced science and technology on the continent, much more is needed to fill the genomic data gap in Africa, notably at the grassroots. More commitment to funding and sponsorship from the Government, private and corporate establishments, and individuals in Africa will also go a long way.

### Building African science to be self-sustainable

An aspect of concern with foreign grants is the continuity of these grants and the adverse regression that science and genomics research in Africa would face if these foreign funding bodies pull out. More so, these funding bodies have their objectives already set, which sometimes might not be Africa’s health priority. The long-term goal would then be to build African research to be self-sustainable through the commercialization of research findings translated into innovations, industrial processes, or tangible products for human use. Wanjiru [43] and John Ayisi [44] examined the role and crucial steps for research commercialization for sustainable development and economic growth in South Africa and Kenya, respectively. Typically, the Bayh-Dole Act of 1980 of the United States established the legal framework for commercializing research developed within university settings by transferring ownership of intellectual property from publicly funded granting agencies to universities [45]. And this sole Act has contributed well over $1.3 trillion to U.S. economic growth and created more than 4.2 million jobs and over 11,000 new startups [46]. Similar models can be adopted and adapted for sustainable science in Africa. In the long term, research capacities should be developed in Africa to build self-sustainable facilities and capacities that can lead the course of total transformation in genomics and the entire science field in Africa.

### Developing capacity for high-end scientific publications and data analysis

The paradox of most research conducted in Africa is that they are not properly documented or not documented at all. For instance, Nigeria has over 170 universities (polytechnics, technical and vocational schools excluded). In 1996/1997, the country produced about 50,000 graduates, out of which 18,805 were reported to be science/science-related graduates [47]. While this figure has progressed geometrically in recent years, Nigeria’s scientific publication stands at 0.3% of global scientific and technical journal articles in 2020, which when pooled with other African countries give less than 1% of scientific publications globally [9].

There is a dire need to develop the capacity of Africans especially young and early career scientists to produce scientific publications that are of international standard. Tertiary institutions in Africa that teach research writing skills are barely a handful and are not enough to bring Africa to par with other continents, hence the need for training, especially from the baccalaureate level is important. Also, bioinformatics skills are needed to analyze and interpret data from genomics research. Without bioinformatics expertise, health and genomics research in Africa is largely going to be suboptimal. There is an enormous need for bioinformaticians in Africa as this field is largely untapped in the region [48]. Consequently, evaluating the interplay of genomics, transcriptomics, and proteomics [49] in the African populace will be essential to better understand any data from genomic research. Bioinformatics and data science research have boundless potential across Africa due to its high level of genetic diversity and the burden of infectious diseases.

### Strategic funding and partnership

Most public institutions with genomic sequencing capabilities are non-profit and have been funded by external bodies on projects they keenly focus on. The fortunate researchers in these institutions represent very few percentages of the African research population. The top funded researchers represent a small fraction (about 2%) of researchers on the continent and are primarily from Agriculture and Health Sciences [42]. This leaves a gap in other disciplines. Private companies like Inqaba Biotec and 54 Gene are contributing their quota to grant African scientists access to their Next Generation Sequencing platforms. Development in sequencing technologies has come a long way, becoming less expensive in recent years. Whole Genome sequencing now costs about $1000 compared to the Human Genome Project, which cost about $3 billion [50]. However, due to no or poor funding, few African researchers can afford to own this infrastructure or pay for such services in a continent where most graduate studies and research (M.Sc and Ph.D.) are self-funded by students who still face the harsh economic realities of the African continent. For context, about 460,065,747 (33%) of the African population are living in extreme poverty (on ≤ $1.90 a day) [51].

## Conclusion

Africa is a genetically diverse continent representing 54 countries with over 3000 ethnic groups. Analysis of large-scale genomic data is a crucial component of precision medicine and has one of the highest potentials to inform disease research and clinical care. Thus, it will benefit the global population and not just Africans if a significant portion of African genome is included in research studies. If the African genomic data is not considered, there is a risk of disease burden in Africa and the world due to population disease re-infection, drug and vaccine inefficacy and non-inclusive clinical trial results. This underrepresented population can serve as a human reservoir of some lethal diseases, which are likely to spread through globalization. Hence, equitable representation of all human populations in genomic studies will give more insight into disease burdens, benefiting all populations worldwide. Hence, the call for action is that stakeholders in the field of genomics in Africa especially governments, private, and corporate bodies and individuals play collaborative roles in developing research and expertise capacities through strategic funding, specialized training, improved partnerships.

## Data Availability

Not applicable to this study.

## Abbreviations

AfSHG: African Society of Human Genetics
ACEGID: The African Centre of Excellence for Genomics of Infectious Diseases
ADR: Adverse drug reactions
AUSAID: Australian Agency for International Development
CYP2D6: Cytochrome P450-Mediated Drug Metabolism Enzyme
CAfGEN: Collaborative African Genomics Network
GDP: Gross Domestic Product
GISAID: Global Initiative on Sharing Avian Influenza Data
GWAS: Genome-wide association studies
Inqaba Biotec: Africa’s Genomics Company
H3Africa: The Human Heredity and Health in Africa Consortium
HGP: Human Genome Project
HIV: Huaman Immunodeficiency Virus
HIC: High-Income Countries
NIH: National Institute of Health
NCD-GHS: Non-Communicable Diseases Genetic Heritage Study
NZAID: New Zealand Agency for International Development
USAID: United States Agency for International Development
WHO: World Health Organization
